# Phylogeography of Rift Valley Fever Virus in Africa and the Arabian Peninsula

**DOI:** 10.1371/journal.pntd.0005226

**Published:** 2017-01-09

**Authors:** Abdallah M. Samy, A. Townsend Peterson, Matthew Hall

**Affiliations:** 1 Biodiversity Institute, University of Kansas, Lawrence, Kansas, United States of America; 2 Entomology Department, Faculty of Science, Ain Shams University, Abbassia, Cairo, Egypt; 3 Institute of Evolutionary Biology, University of Edinburgh, Edinburgh, United Kingdom; 4 Centre for Immunity, Infection and Evolution, University of Edinburgh, Edinburgh, United Kingdom; 5 Department of Infectious Disease Epidemiology, Imperial College London, London, United Kingdom; Aix Marseille University, Institute of Research for Development, and EHESP School of Public Health, FRANCE

## Abstract

Rift Valley Fever is an acute zoonotic viral disease caused by Rift Valley Fever virus (RVFV) that affects ruminants and humans in Sub-Saharan Africa and the Arabian Peninsula. We used phylogenetic analyses to understand the demographic history of RVFV populations, using sequence data from the three minigenomic segments of the virus. We used phylogeographic approaches to infer RVFV historical movement patterns across its geographic range, and to reconstruct transitions among host species. Results revealed broad circulation of the virus in East Africa, with many lineages originating in Kenya. Arrival of RVFV in Madagascar resulted from three major waves of virus introduction: the first from Zimbabwe, and the second and third from Kenya. The two major outbreaks in Egypt since 1977 possibly resulted from a long-distance introduction from Zimbabwe during the 1970s, and a single introduction took RVFV from Kenya to Saudi Arabia. Movement of the virus between Kenya and Sudan, and CAR and Zimbabwe, was in both directions. Viral populations in West Africa appear to have resulted from a single introduction from Central African Republic. The overall picture of RVFV history is thus one of considerable mobility, and dynamic evolution and biogeography, emphasizing its invasive potential, potentially more broadly than its current distributional limits.

## Author Summary

Rift Valley Fever is an acute zoonotic viral disease caused by Rift Valley Fever virus (RVFV) that affects ruminants and humans in Sub-Saharan Africa and the Arabian Peninsula. We used phylogenetic approaches that take sampling date into account to understand the demographic history of RVFV populations, using sequence data from the three minigenomic segments of the virus. We found evidence of broad circulation of the virus in East Africa, with many lineages originating in Kenya, with single and multiple introductions of RVFV among countries. The overall picture of RVFV history is thus one of considerable mobility, and dynamic evolution and biogeography, emphasizing its invasive potential, potentially more broadly than its current distributional limits.

## Introduction

Rift Valley Fever (RVF) is an acute zoonotic viral disease caused by RVF virus (RVFV; *Phlebovirus*, family *Bunyaviridae*) that affects both large mammals and humans, and that is transmitted by *Aedes* and *Culex* mosquitoes [1]. It causes high mortality and abortions in ruminants [2]; while infections in humans are characterized by febrile illness, followed by hemorrhagic fever, encephalitis, and ocular disease, and can lead to death [2]. It is endemic in Sub-Saharan Africa, being first isolated in Kenya in 1930 [3]. Outbreaks were limited to that region until 1977–1978, when the virus spread to Egypt [4]. In 1993, southern Egypt suffered a further outbreak, in which 600–1500 human infections were reported [5]. Periodic RVFV epizootics and epidemics have been associated with above-average rainfall and other environmental factors that result in dramatically increased mosquito populations [6,7].

A recurrence of RVF in East Africa was reported in 1997–1998 [8]. In 1987, the first West African epidemic occurred in Senegal and Mauritania during flooding in the lower Senegal River area [9]. The first outbreaks outside Africa occurred in 2000, in Saudi Arabia and Yemen [10]. In 2000–2010, outbreaks were reported in Sudan, Kenya, Tanzania, Somalia, Senegal, Mauritania, and Swaziland, with incidence rates higher than in the 1978 Egyptian epidemic [11–14]. RVFV has not apparently become endemic outside Africa, but seropositive animals have been detected in Saudi Arabia [15]. Climate conditions are appropriate for incursions of RVFV elsewhere in the Middle East, Europe, and beyond [10,16].

RVFV has been isolated from both livestock and mosquitoes [17,18]. The virus is maintained in mammal host species, including cattle, sheep, goats, and camels, in which infections have been reported [18,19]. RVFV is transmitted via several routes: mosquitoes serve as vectors in most cases, but direct transmission through aerosol and contact with abortion products are other routes [20]. RVFV is also capable of persisting in the environment for long periods between epidemics [21], facilitated by vertical transmission among mosquitoes [22].

The RVFV genome is organized in three negative-sense, single-stranded RNA segments termed large (L), medium (M), and small (S), with a total genome length of 11.9 kb. The large segment (∼6.4 kb) encodes the RNA-dependent RNA polymerase [23]; the M segment (∼3.2 kb) encodes envelope glycoproteins *G*_*n*_ and *G*_*c*_, plus two accessory proteins, NSm and the 78-kDa protein [24]. The S ambisense segment (∼1.7 kb) encodes for nucleoprotein (NP; 27 kDa) and non-structural protein (NSs; 31-kDa). Previous studies have sequenced the three virus segments from diverse strains circulating in outbreaks across Africa and Saudi Arabia [25].

Historical movements of RVFV among countries raise concerns about possible appearance of RVFV in new regions [16]. Here, we aim to derive a detailed picture of RVFV phylogeny based on analysis of sequences of the three segments. We used phylogeographic approaches to examine mobility patterns of virus lineages across the virus’ geographic distribution.

## Materials and Methods

Data used in this analysis represent all RVFV strains deposited in GenBank (as of August 2014; http://www.ncbi.nlm.nih.gov/nuccore), and include full sequences of the L, M, and S segments. If two or more records were available from the same isolate, we included the more recently sequenced version in analyses. Sequences for which the GenBank metadata listed no country of origin were excluded from those analyses. Sequences were aligned using the MUSCLE plugin [26] in the MEGA 6 software [27].

We used JModelTest [28] to identify the best-fitting nucleotide substitution model for each of the segments separately. A molecular clock-based phylogenetic analysis was performed for each segment separately in BEAST [29], using the best-fitting nucleotide substitution model for each segment, an uncorrelated lognormal relaxed molecular clock [30], and a GMRF Bayesian skyride tree prior [31]. Because variation among virus sampling dates (i.e., 1944–2010) is of meaningful magnitude relative to the time to most recent common ancestor of the clade in question [32,33], the temporal information associated with sampling each isolate had to be taken into consideration [30,34]. Sampling dates were used as prior information to calibrate the tree, estimate ages of different RVFV lineages, and infer evolutionary history of the strains [29]. An uncorrelated lognormal relaxed molecular clock was used in light of its high accuracy and precision to infer temporal information into molecular phylogeny [30]. Codon positions 1 and 2, and codon position 3, were treated as two separate partitions in the alignment. Multiple Monte Carlo Markov Chain (MCMC) runs of 10^8^ states (the first 10% was discarded as burn-in) were combined to achieve estimated sample sizes of at least 250 for all numerical model parameters.

The posterior set of trees from each of the three initial BEAST analyses was used as an empirical tree set for a discrete-trait phylogeography analysis [35]. We assumed an asymmetrical rate matrix. For each tree sampled from the MCMC, Markov Jumps procedure [36] was used to reconstruct a stochastic realization of the between-country diffusion process; results were summarized over the entire posterior distribution by calculating median numbers of transitions between each pair of countries and the posterior probability that at least one transition occurred.

As the full dataset consisting of every available RVFV sequence in GenBank is a very uneven sample, we wished to confirm that our results were robust to this unevenness. Hence, we repeated these analyses using datasets produced by randomly removing sequences from each alignment until they contained no more than one sequence from isolates obtained in a single country during a single year. For each segment, the molecular clock and phylogeography procedure was then repeated as described above, for these smaller datasets. The smaller size of these datasets led to poor MCMC convergence when the JModelTest-identified substitution models were used, so we employed the SRD06 [37] model instead. All other settings in BEAST were identical.

Reassortment between RFV segments was investigated by concatenating the alignments for the three segments, retaining only sequences from isolates for which at least two segments were available. We used the RDP method implemented in the program RDP4 [38] to identify recombination breakpoints in the concatenated alignment. A reassortment event was implied where these breakpoints coincided with segment boundaries.

## Results

### Sequence data

A total of 155 S, 99 M, and 97 L minigenomic segments of RVFV sequence data were available from Genbank (Table 1). Sequences had lengths of 1689–1692 base pairs (bp) for S, 3871–3885 bp for M, and 6397–6404 bp for L. These sequences represented RVFV strains from 18 countries across Africa plus Saudi Arabia (Table 1). Saudi Arabian strains were represented by sequences for two S segments, one M segment, and one L segment only. Full details of the sequence data are available via Figshare (https://figshare.com/s/1efd1db044bcaa9e35a9), including information for sequence accession, sequence length, country of origin, and isolation host.

**Table 1:** Countries and dates of sampling available for the complete sequences of the small, medium, and large segments of RVFV strains across Africa, and Arabian Peninsula.

Country	No. of isolates			Date range (Years)*
	Small	Medium	Large	
Angola	1	0	0	1985
Burkina Faso	2	1	1	1983
Central African Republic	8	6	6	1969–1985
Egypt	9	7	10	1977–1994
Guinea	3	2	2	1981–1984
Kenya	63	45	42	1951–2007
Madagascar	14	11	10	1979–2008
Mauritania	5	4	4	1987–1988
Mayotte	2	2	2	2008
Namibia	1	0	0	2004
Saudi Arabia	2	1	1	2000
Senegal	1	0	0	1981
Somalia	1	0	0	1998
South Africa	10	4	4	1951–2008
Sudan	13	5	4	2007–2010
Uganda	2	1	1	1944–1955
United Republic of Tanzania	4	4	4	2007
Zambia	1	0	0	1985
Zimbabwe	13	6	6	1970–1979
All sequences	155	99	97	1944–2010

* The date range represents the range of sampling RVFV strains to which the sequence is identified. When a single sequence identified in a country, the date range represent the date of sampling for this single strain.

### Molecular clock and skyride analysis of RVFV strains

**Fig 1:**
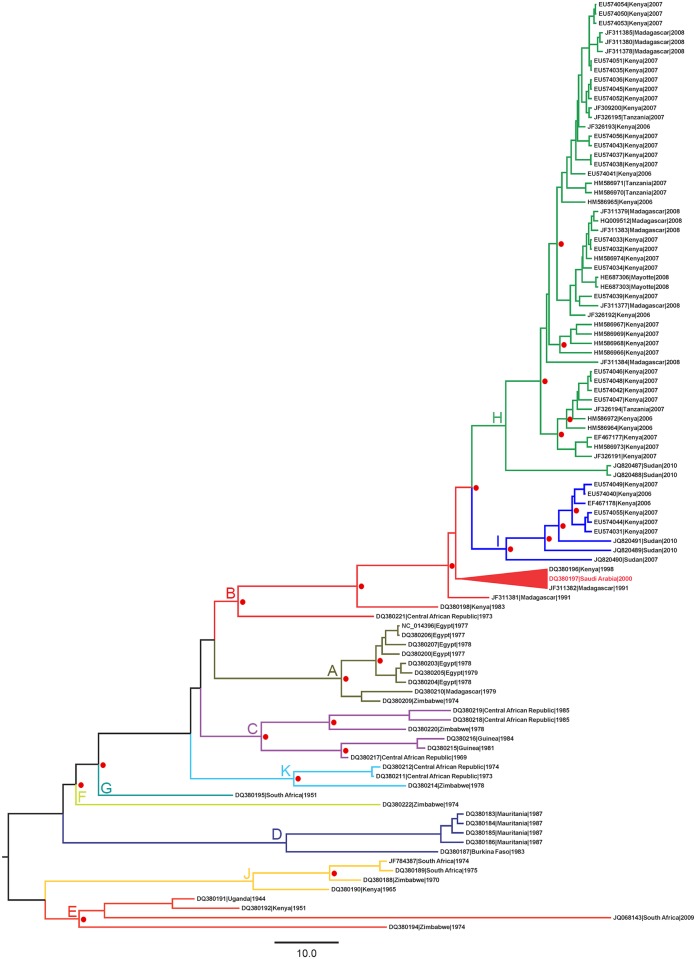
Maximum Clade Credibility tree based on all sequences of the medium minigenomic segment (M) of RVFV isolates in the study. NCBI accession number, country, and date of sampling are presented at the tree tips. Tree branches are colored and labelled alphabetically by lineage (A to K). Lineage nomenclature is from Bird et al. [32]. The red triangle identifies the clade containing isolates from both Saudi Arabia and Africa. Clades with posterior probability >0.9 are labelled with red circles.

The nucleotide substitution models with the lowest Akaike Information Criterion scores identified by JModelTest were TPM2uf+I+G for the S segment, and GTR+I+G for both M and L. The maximum clade credibility (MCC) tree for the M segment is presented in Fig 1; trees for the other two segments can be found in the supplementary materials (S1 and S2 Files). Lineages previously identified and discussed by Bird et al. [32] are indicated on the trees.

Estimated posterior mean nucleotide substitution rates were 3.6392 x 10^−4^, substitutions per site per year, with a 95% highest posterior density (HPD) interval of 2.8114 × 10^−4^ to 4.5813 × 10^−4^, for the S segment, 3.7774 × 10^−4^ (2.7391 × 10^−4^ to 4.8902 × 10^−4^) for M, and 2.7310 × 10^−4^ (1.9289 × 10^−4^ to 3.6677 × 10^−4^) for L. The posterior mean calendar year of the most recent common ancestor (TMRCA) of all isolates was 1929 (1920–1937) for S, 1914 (1897–1928) for M, and 1909 (1888–1927) for L.

**Fig 2:**
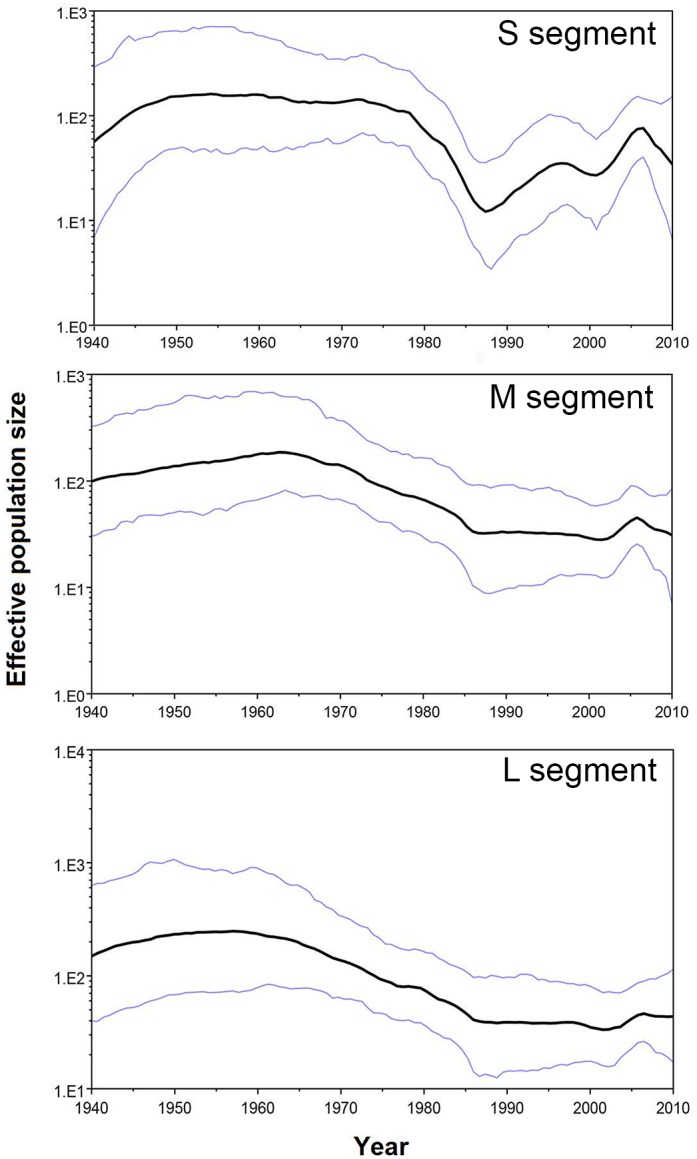
Gaussian Markov Random Field (GMRF) Bayesian skyride plots for all three RVFV segments, representing the relationship between reconstructed effective population size and calendar year. Blue lines show the boundaries of the 95% highest posterior density interval.

Skyride plots reconstructing temporal variation in RVFV genetic diversity are presented in Fig 2 for all three segments. All three indicate a peak in diversity around the middle of the twentieth century, followed by a decline and levelling off, with a subsequent increase in the reconstruction for the S segment.

The downsampled datasets consisted of 49 sequences for S, 33 for M, and 32 for L. For L, the posterior mean substitution rate was 2.406 × 10^−4^ substitutions/site/year (1.106 × 10^−4^ to 4.4065 × 10^−4^) and the posterior mean TMRCA was 1870 (1810–1919). For S, the rate was 3.636 × 10^−4^ substitutions/site/year (2.058 × 10^−4^ to 5.248 × 10^−4^) and the TMRCA was 1914 (1893–1935). These rate estimates are consistent with those estimated from the full dataset, although the TMRCA point estimates are rather different; we noted a clear loss of precision in using the smaller alignments and considerable HPD interval overlap. For M, however, the posterior mean rate was 2.74 × 10^−4^ substitutions/site/year (1.965 × 10^−4^ to 3.573 × 10^−4^) and the posterior mean TMRCA was 1881 (1844–1907). The results of the posterior mean nucleotide substitution rates and TMRCA from both full and downsampled datasets are summarized in supplementary materials (S3 File). The skyride plots for the downsampled analyses (S4 File) did not indicate that the inclusion of all GenBank sequences had any major effect on the demographic reconstructions.

### Phylogeography of RVFV strains

**Fig 3:**
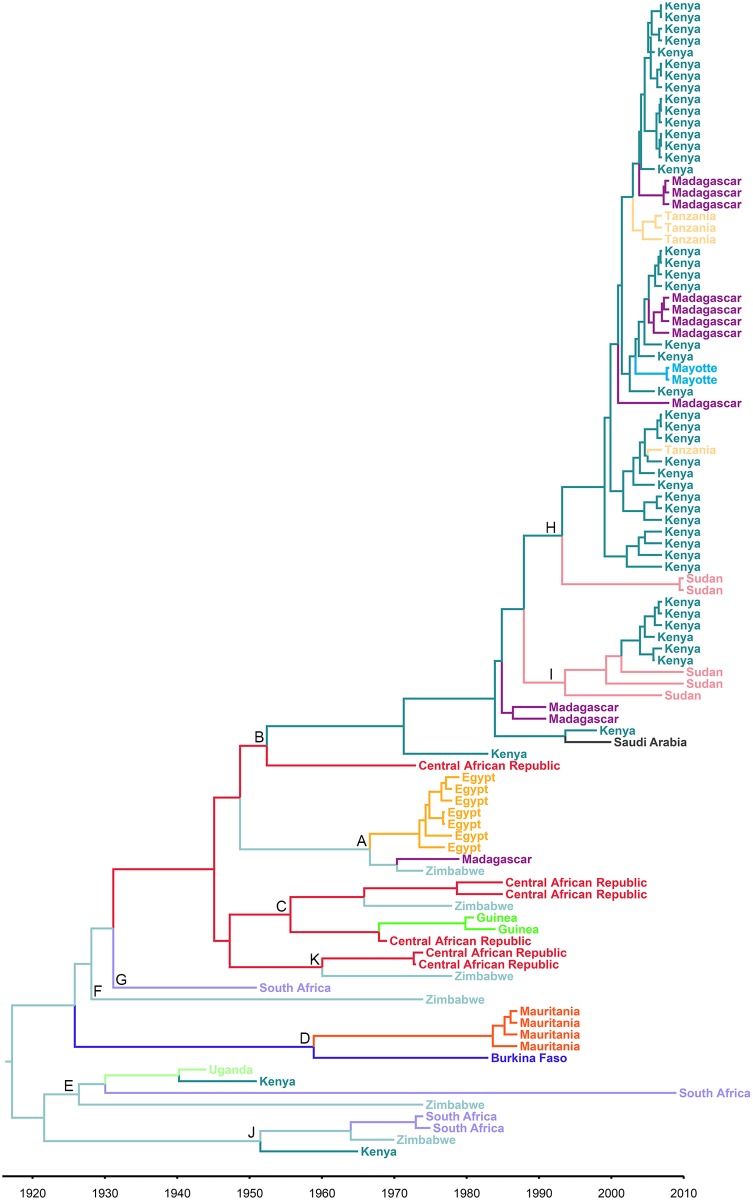
Maximum Clade Credibility tree based on all sequences of the medium minigenomic segment (M) of RVFV isolates in the study. Country of origin is indicated by color of the tree branches and branch tips. An online version of the tree is available via https://figshare.com/s/1efd1db044bcaa9e35a9.

Fig 3 presents the MCC phylogeny for the M segment, this time with branches colored by highest posterior probability of location; trees for the other two segments are in the supplementary materials (S5 and S6 Files). The tree shows that strains from Saudi Arabia belong to the same lineage (B) as those from Kenya in 1998 and Madagascar in 1991. Most strains from West Africa (Mauritania and Burkina Faso) are part of lineage D, but those from Guinea are in lineage C, which is otherwise recorded in the Central African Republic and Zimbabwe.

**Fig 4:**
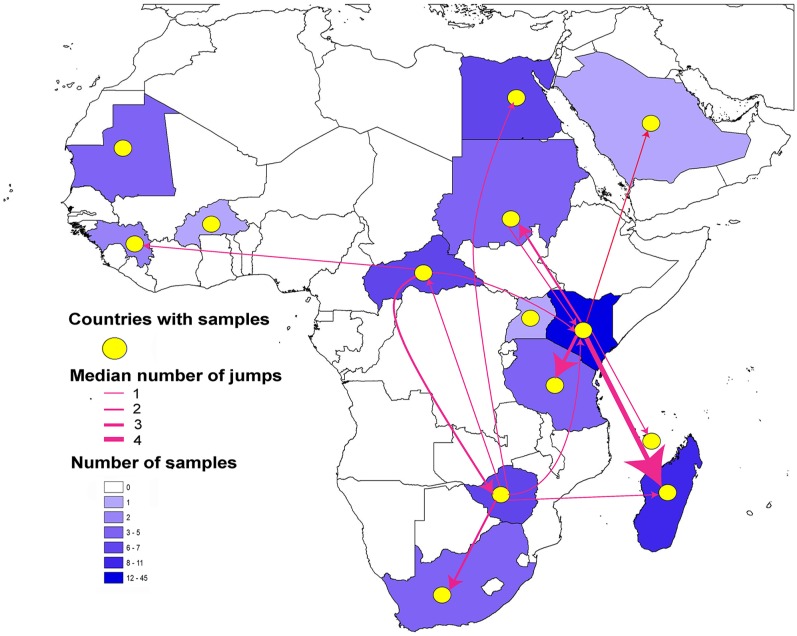
Connectedness of countries with Rift Valley Fever outbreaks based on Markov Jumps reconstruction using the medium minigenomic segment (M) of RVFV isolates in the study. Connections between countries are presented as lines with arrows to refer to the direction of movement. Line thickness identifies the median number of jumps between each country pair.

The diffusion patterns for viral lineages reconstructed using Markov Jumps are presented in Fig 4 & S7–S11 Files; the complete results of the Markov Jumps analysis for all countries are available via Figshare (https://figshare.com/s/1efd1db044bcaa9e35a9). The reconstruction using all three segments revealed that the highest median number of jumps was from Kenya to other countries in East Africa; movements of RVFV lineages from Kenya to Tanzania are particularly well supported (posterior probability >0.9). Hence, arrival of the virus in Tanzania in 2007 was probably related to a single introduction event from Kenya. Introductions of RVFV to Madagascar came in three waves: the first from Zimbabwe, and the second and third from Kenya.

The two major outbreaks in Egypt since 1977 originally may have been the result of a long-distance introduction from Zimbabwe, as they are closely related to strains from that country in 1974. The Markov Jumps reconstruction revealed a possible transition from Kenya to Mayotte, with posterior probabilities ranging from 0.83 to 0.96 in different genomic segments. All sequences from Sudan came from a single outbreak in 2007–2010, and were closely related to isolates from outbreaks occurring in other East African countries since 2007; the reconstruction suggested direct movement of the virus from Kenya to Sudan (posterior probability >0.7).

Markov Jumps analysis of M and L segments indicated virus introduction from Zimbabwe to the Central African Republic (posterior probabilities 0.51 and 0.64, respectively). Other movement patterns inferred included transitions from Zimbabwe to South Africa (posterior probabilities 0.59, 0.68, and 0.78 for S, M, and L, respectively). M and L segments revealed a single transition into West Africa, from the Central African Republic to Guinea.

The M and L segments suggested a single introduction from Kenya to Saudi Arabia, with a posterior probability >0.79 for both segments. Movement of the virus between Kenya and Sudan occurred in both directions, with posterior probabilities of >0.7. Similarly, transitions between Central African Republic and Zimbabwe were reconstructed in both directions (posterior probabilities 0.57, 0.79, and 0.69 for S, M, and L, respectively, for Central African Republic to Zimbabwe; posterior probabilities 0.62, 0.51, and 0.64 for Zimbabwe to Central African Republic, for S, M, and L, respectively). The S segment revealed information about movements involving countries for which it was the only segment available: from Kenya to Somalia (posterior probability 0.77), Burkina Faso to Mauritania (posterior probability 0.80), Burkina Faso to Senegal (posterior probability 0.78), Kenya to Central African Republic (posterior probability 0.91), and Kenya to South Africa (posterior probability 0.91). Results of phylogeographic analyses for the downsampled datasets were similar with the results from those reported above (S12–S14 Files).

### Reassortment and recombination events of RVFV

The full concatenated alignment was 11,999 bp in length, with the S segment occupying positions 1–1695, the M segment positions 1696–5595, and the L segment positions 5596–19999. The RDP algorithm identified two events that suggest segment reassortment. The first suggested that strain 2007000608 is a reassortment of the L and S segments of 2007000234 and the M segment of 2007001811. All three isolates were sampled in Kenya in late 2006 or early 2007 [32]. However, the GenBank records for the M segments of 2007000608 and 2007001811 are identical, which raises the possibility of a database error. The estimated breakpoints were at positions 1581 (95% confidence interval 1238–2043) and 5565 (5391–5917), strongly suggesting agreement with segment boundaries.

The second event suggested that the Tanzanian isolate TAN/Tan-001/07 was a reassortment of the M segment from another Tanzanian isolate, TAN/Dod-002/07, and the L and S segments from the Kenyan isolate 2007001564. All three were sampled in early 2007 [39] and no two segments were identical. RDP identified a number of small recombination events, but none involved a longer genomic region. Again, the breakpoints at positions 1919 (1385–2202) and 5441 (5285–5917) were consistent with reassortment. RDP identified four other potential recombination events whose breakpoints did not coincide with segment boundaries in the concatenated alignment, and therefore were not suggestive of reassortment; see supplementary file for details (S15 File).

## Discussion

This study used up-to-date phylogenetic approaches [35] to investigate the ancestry of RVFV strains across Africa and Saudi Arabia, and to study virus movements and host transitions. Sequences for the three segments of the RVFV genome were available for strains sampled over a span of 66 years (1944–2010). RVFV minigenomic segments showed differences in amounts of genetic change and time scale [33]. These minigenomic segments have previously been used to derive phylogenetic and ecological insights regarding RVFV circulation in Africa and the Arabian Peninsula [32,33]. These analyses used phylogenetic approaches similar to ours; however, our analysis takes advantage of a discrete-traits phylogenetic analysis using Markov Jumps to infer the history of between-country and between-host movements.

The substitution rate estimates in our study were similar to those in previous studies [33,43]. Other studies have reported higher rates [32,44]: for example, estimates from Aradaib *et al*. [44] were 4.20 × 10^−4^, 5.06 × 10^−4^, and 4.29 × 10^−4^ substitutions per site per year for S, M, and L, respectively; the 95% HPD intervals reported in that paper overlap with ours. These differences might be a result of the different datasets used: our work should have better resolution because the dataset is larger and more diverse. The Aradaib *et al*. [44] study was also limited to only two lineages of the 11 in our analysis, and covered only strains from Kenya, Sudan, Madagascar, and Zimbabwe. Interestingly, Freire *et al*. [33] found a lower evolutionary rate in the large minigenomic segment, suggesting a distinct evolutionary history in the three segments; our results corroborated this finding [33].

Previous studies reported earlier TMRCA estimates than ours. Bird *et al*. 2007 [32] estimated the mean TMRCA as 1891 for the S segment, 1882 for M, and 1887 for L. These differences could be a result of our larger dataset, and reflect higher estimates of substitution rates; again, their HPD estimates overlap with ours. As the HPD intervals for the TMRCAs of the three segments in our analysis all overlap, the difference in point estimates likely reflects statistical uncertainty only. Our TMRCA estimates for all segments agreed well with the first report of RVFV in 1930 in Kenya [3].

The steady decline in RVFV genetic diversity since the 1970’s was previously reported [33], in an analysis that considered most of our samples. A possible explanation for the decline centers on the vaccination and control measures implemented on a large scale from 1969 to 1979 [45]. In all, 35.2 million vaccines were provided to Zimbabwe, South Africa, Namibia, Israel, and Egypt in response to large RVF outbreaks [46].

Discrete-traits phylogenetic approaches have some limitations [34]. In our case, these limitations are associated with the nature of virus sampling across its range, as sampling is generally unbalanced. This point suggests that some aspects of our results should be interpreted with caution. For example, country was used as indication of location in our analyses, which is quite coarse for some of the spatial phenomena that we would like to reconstruct. As more sequence data become available, it should be possible to develop finer-resolution views.

In Sub-Saharan Africa, RVFV appears to be spread by movement of viremic livestock between countries [47,48], or though introduction of infected mosquitoes to neighboring countries [16,48]. We used Markov Jumps to infer possible introduction events and movement routes of RVFV. Two types of RVFV movements can be considered: short- and long-distance jumps [32,49,50]. Inferred movements between distant countries may omit the effects of unsampled lineages in countries on the route between them. For example, studies attributed the 1977–1978 epidemics in Egypt to viral introductions from Sudan [51,52], but our study saw strong support for Zimbabwe as a country of origin for Egyptian strains. This result suggests that, although Zimbabwe was the sampled origin for these lineages, they travelled north over Sudan to Egypt; all available Sudanese sequences came from more recent outbreaks in 2007–2010, which presumably originated in Kenya, and are genetically distant from Egyptian strains in the outbreak of the 1970s [44]. With no earlier Sudanese sequences available [53,54], this analysis could not find an origin in Sudan, and hence tracked lineages back to Zimbabwe; this result should thus not be taken to indicate that the hypothesis of a Sudanese origin for Egyptian epidemics is incorrect.

The RVFV strain identified from the Arabian Peninsula in 2000 was embedded in lineage B with strains from Kenya, suggesting that this virus originated from Kenyan epizootics in 1997–1998. The outbreak was driven by floods and heavy rains along the Saudi Arabia-Yemen border in the Al Humayrha region, where the first cases were reported [55], and where it was maintained by *Culex tritaeniorhynchus* [56].

RVFV strains from West Africa fell in two lineages (C & D): one included samples from Guinea and another that included samples from Burkina Faso, Mauritania, and Senegal. Our results suggest possible introduction of RVFV to Guinea from the Central African Republic, and that the outbreak in Mauritania in 1987 had its origin in lineages that were in Burkina Faso in 1983. The route of introduction from East Africa to West Africa more fundamentally is still unclear. Our analysis suggested interesting patterns for outbreaks in Mauritania and Egypt, in comparison to recent outbreaks in Kenya, with single viral introductions to Mauritania and Egypt, but multiple origins for the 2007–2010 outbreaks in Kenya.

These latter phylogeographic analyses revealed the overall picture of RVFV history and migration across Africa and Arabian Peninsula. Early RVFV strains were restricted to Sub-Saharan Africa; however, the virus was later identified from several parts across North and West Africa. The considerable mobility and dynamic distribution of the virus allowed spread and invasion of the virus to new regions, including in the Arabian Peninsula. Understanding the movement patterns between countries represents one of the major reasons why we set out to understand the detailed picture of the virus evolution and biogeography.

Previous studies identified evidence of reassortment events among RVFV segments [32,33,41,42]. Our phylogenetic analyses showed incongruences in the topologies of the three minigenomic segments of RVFV, these incongruences could result from genetic reassortments. Final analyses to investigate the reassortment and recombination events were based on the RDP algorithm: all strains potentially involved in the reassortment events were from East Africa. This possibility of reassortment raises two important concerns in regard to the potential of RVFV epidemics and its pathogenicity, although detailed studies with which to answer these questions are lacking. Successful reassortment under natural selection requires (1) co-occurrence of at least two different strains in the same host, area, and time; and (2) the reassorted strain would have to be virulent and able to infect a host. The two strains detected as possible reassortments in this study were from humans and bovines, and not restricted to a single host [42]. Although previous studies revealed no evidence of recombination on large scales in RVFV [40,41], this study also suggests that small recombination events may indeed occur, although none involving long gene regions. Reassortment and recombination represent key potential mechanisms to promote emergence of novel strains of RVFV that may be more virulent and can infect broad ranges of hosts, and locations. Further studies investigating possible influences of reassortment events in RVFV evolution across Africa and Arabian Peninsula are much needed.

Our results assist the RVFV control program across its range in three ways. (1) The study shows possible movement and migration of the virus among endemic areas; as such, this study allows tracking virus movement among countries, which may inform transportation guidelines for animal shipments. (2) Our calculations of evolutionary rates and TMRCAs offer a more detailed picture of the temporal nature of successive outbreaks of RVFV. Finally, (3) possible reassortment and recombination events reported herein raise important questions regarding control programs related to novel strains of RVFV across the potential geographic range, attenuated vaccination efficacy, and changing virus pathogenicity. Future studies should collect samples more systematically, and on a much finer scale with respect to location and host, to give a more detailed picture of migratory patterns of RVFV across the continent for a comprehensive study including reconstruction of both geographical and between-host transmission.

## Supporting Information

S1 File MCC tree based on the small minigenomic segment (S) of RVFV isolates. Accession number, country, and date of sampling are presented at the tree tips. Tree branches are colored and labelled alphabetically by lineage (A to K). Lineage nomenclature is from Bird et al. [32]. The red triangle identifies the clade containing isolates from both Saudi Arabia and Africa. Clades with posterior probability >0.9 are labelled with red circles.
(TIFF)

S2 File MCC tree based on all sequences of the large minigenomic segments (L) of RVFV isolates in the study. Accession number, country, and date of sampling are presented at the tree tips. Tree branches are colored and labelled alphabetically by lineage (A to K). Lineage nomenclature is from Bird et al. [32]. The red triangle identifies the clade containing isolates from both Saudi Arabia and Africa. Clades with posterior probability >0.9 are labelled with red circles.
(TIFF)

S3 File Substitution rate and TMRCA estimates of RVFV based on two datasets of sequences; one included all sequences in GenBank, and downsampled datasets representing the random subset from the full sequences. (PDF)

S4 File Bayesian skyride plot, representing the relationship between effective population size and time in years and based on the downsampled datasets. Blue lines show the boundaries of the 95% highest posterior density interval.
(PDF)

S5 File MCC tree based on the small minigenomic segment (S) of RVFV. Country of origin is indicated by color on the tree branches and branch tips. An online version of the tree is available via https://figshare.com/s/1efd1db044bcaa9e35a9.
(TIFF)

S6 File MCC tree based on all sequences of the large minigenomic segment (L) of RVFV isolates in the study. Country of origin is indicated by color on tree branches and branch tips. An online version of the tree is available via https://figshare.com/s/1efd1db044bcaa9e35a9.
(TIFF)

S7 File Connectedness of countries with Rift Valley Fever outbreaks based on Markov Jumps reconstruction using the S sgement of RVFV isolates in the study. Connections between countries are presented as lines with arrows to refer to the direction of movement. The connected countries are countries with non-zero median transition frequencies. Line thickness identifies the median number of jumps between each country pair.
(TIFF)

S8 File Connectedness of countries with Rift Valley Fever outbreaks based on Markov Jumps reconstruction using the L segment of RVFV isolates in the study. Connections between countries are presented as lines with arrows to refer to the direction of movement. The connected countries are countries with non-zero median transition frequencies. Line thickness identifies the median number of jumps between each country pair.
(TIF)

S9 File Animated visualization of diffusion patterns of RVFV across Africa and Arabian Peninsula based on all sequences from the S segment. The video is also available also via Figshare (https://figshare.com/s/1efd1db044bcaa9e35a9).
(MP4)

S10 File Animated visualization of diffusion patterns of RVFV across Africa and Arabian Peninsula based on all sequences from the M segment. The video is also available via Figshare (https://figshare.com/s/1efd1db044bcaa9e35a9).
(MP4)

S11 File Animated visualization of diffusion patterns of RVFV across Africa and Arabian Peninsula based on all sequences from the L segment. The video is also available via Figshare (https://figshare.com/s/1efd1db044bcaa9e35a9).
(MP4)

S12 File Results of the Markov Jumps analysis of the S segment using the downsampled dataset of RVFV isolates in the study. (CSV)

S13 File Results of the Markov Jumps analysis of the M segment using the downsampled dataset of RVFV isolates in the study. (CSV)

S14 File Results of the Markov Jumps analysis of the L segment using downsampled dataset of RVFV isolates in the study. (CSV)

S15 File Results of reassortment and recombination analysis using RDP algorithm. (CSV)
