# Supplementary figures and images for: Phylogeography of Rift Valley Fever Virus in Africa and the Arabian Peninsula
Source: PLoS Negl Trop Dis. 2017 Jan 9;11(1):e0005226. doi: 10.1371/journal.pntd.0005226 (PMC5221768; doi:10.1371/journal.pntd.0005226)

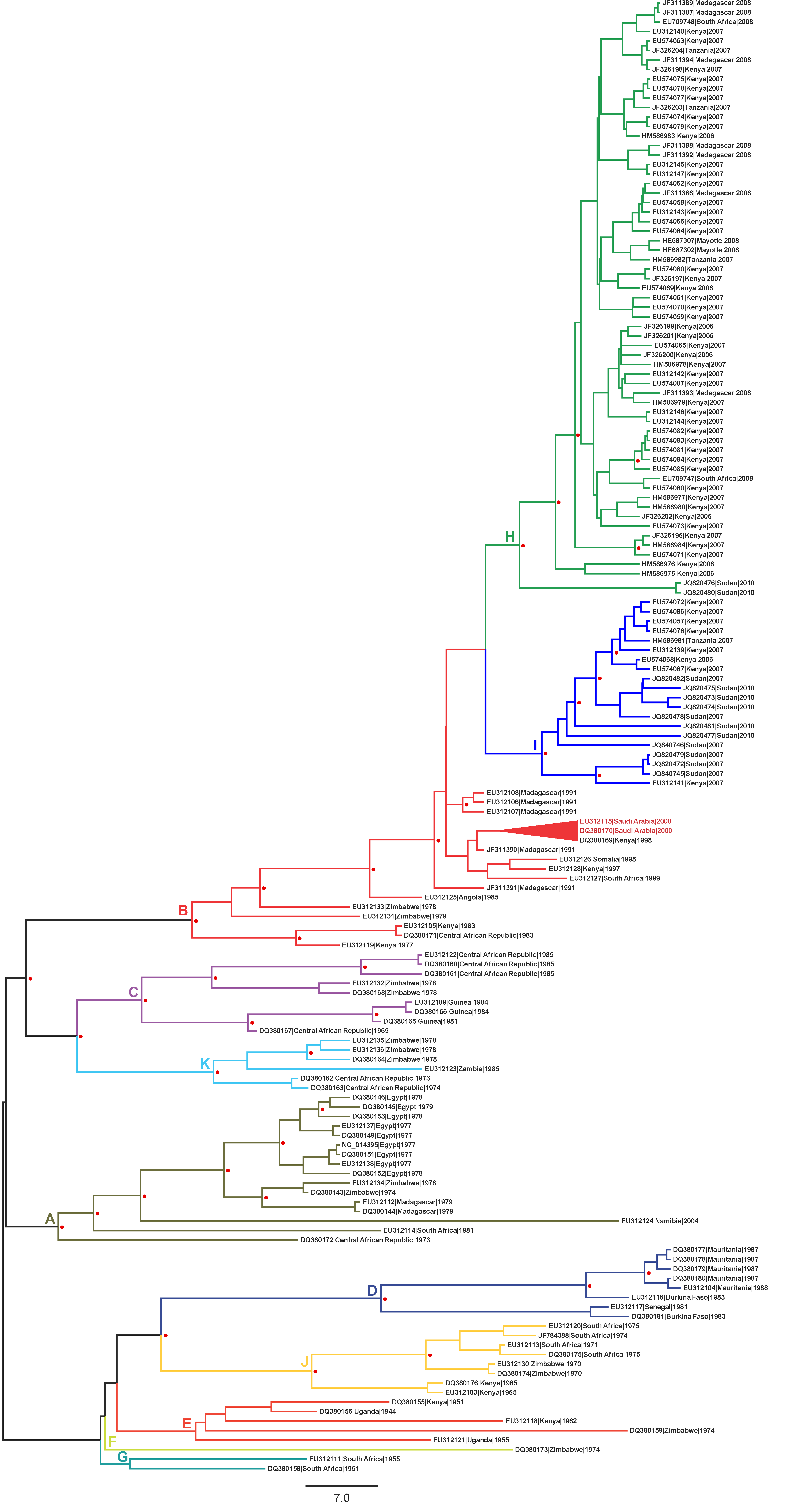

Supplement: S1 File — Accession number, country, and date of sampling are presented at the tree tips. Tree branches are colored and labelled alphabetically by lineage (A to K). Lineage nomenclature is from Bird et al. [32]. The red triangle identifies the clade containing isolates from both Saudi Arabia and Africa. Clades with posterior probability >0.9 are labelled with red circles. (TIFF) [file pntd.0005226.s001.tiff]

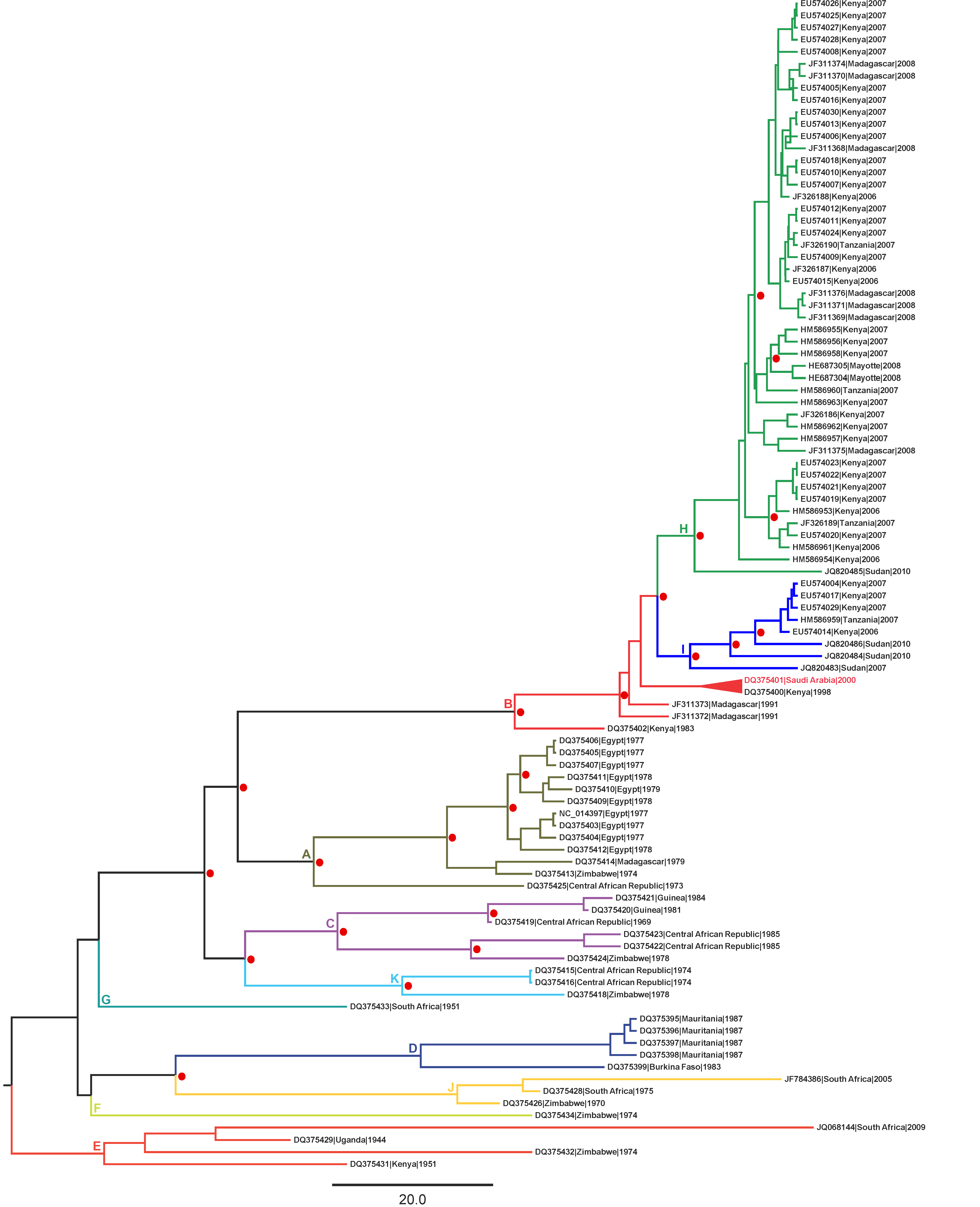

Supplement: S2 File — Accession number, country, and date of sampling are presented at the tree tips. Tree branches are colored and labelled alphabetically by lineage (A to K). Lineage nomenclature is from Bird et al. [32]. The red triangle identifies the clade containing isolates from both Saudi Arabia and Africa. Clades with posterior probability >0.9 are labelled with red circles. (TIFF) [file pntd.0005226.s002.tiff]

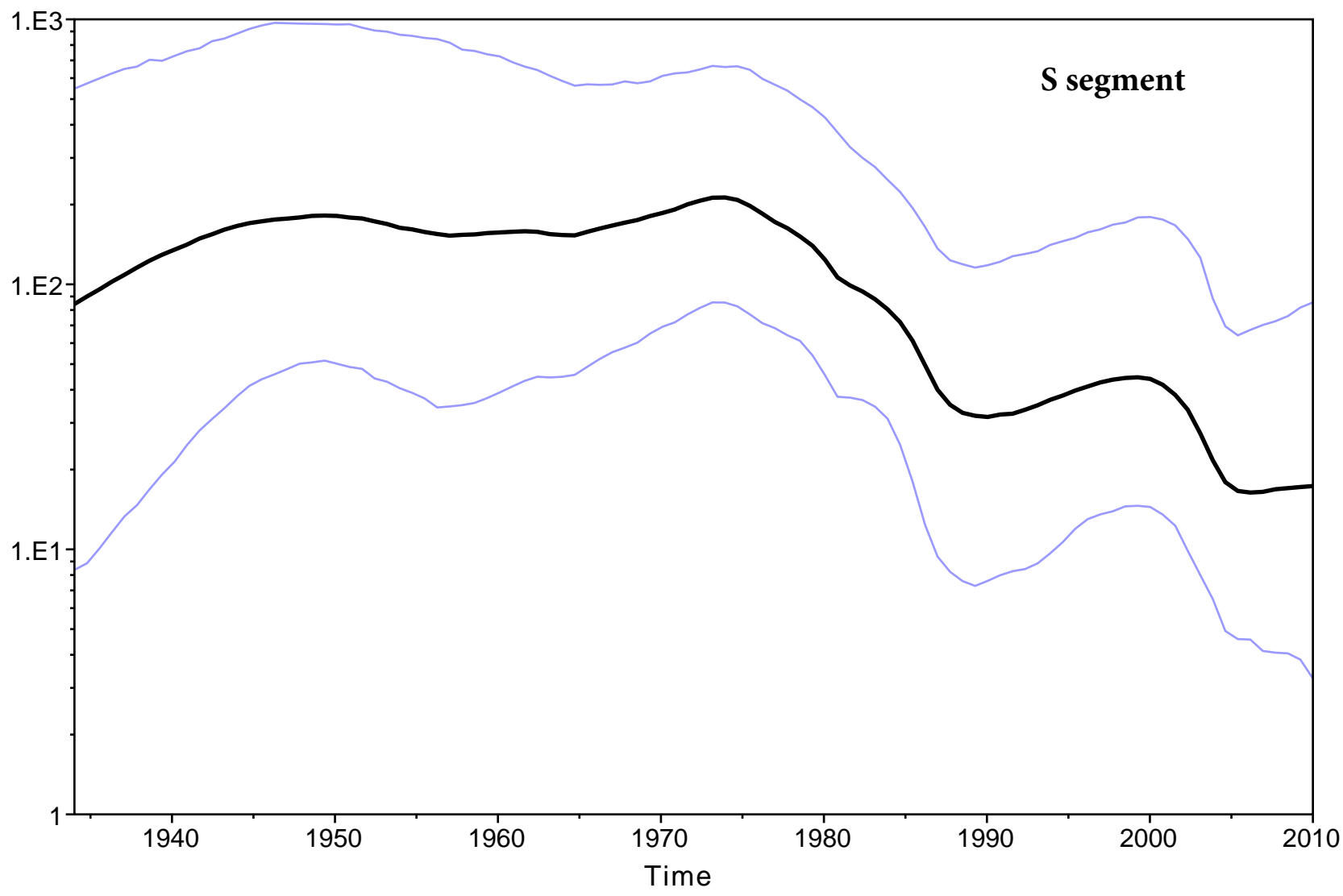

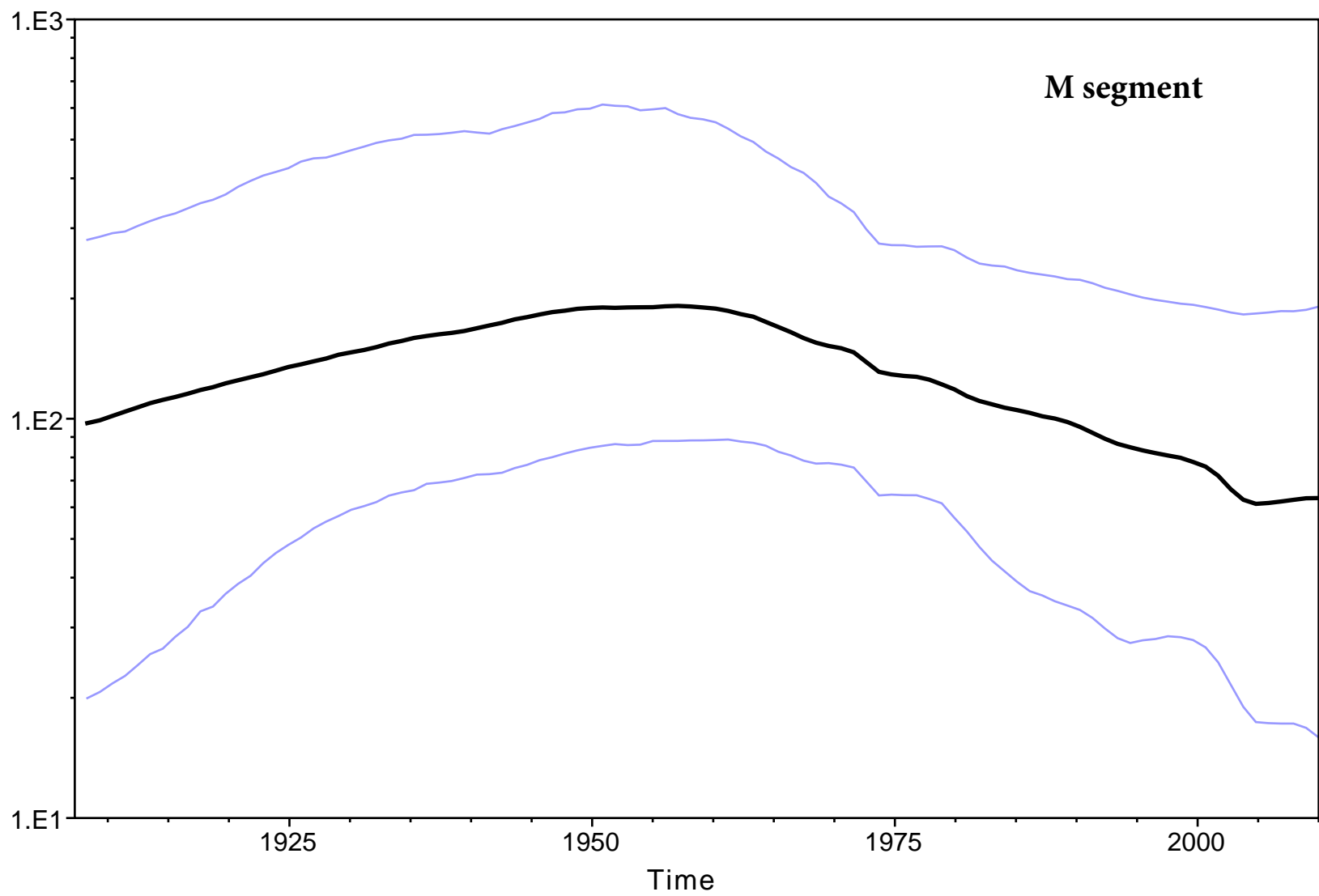

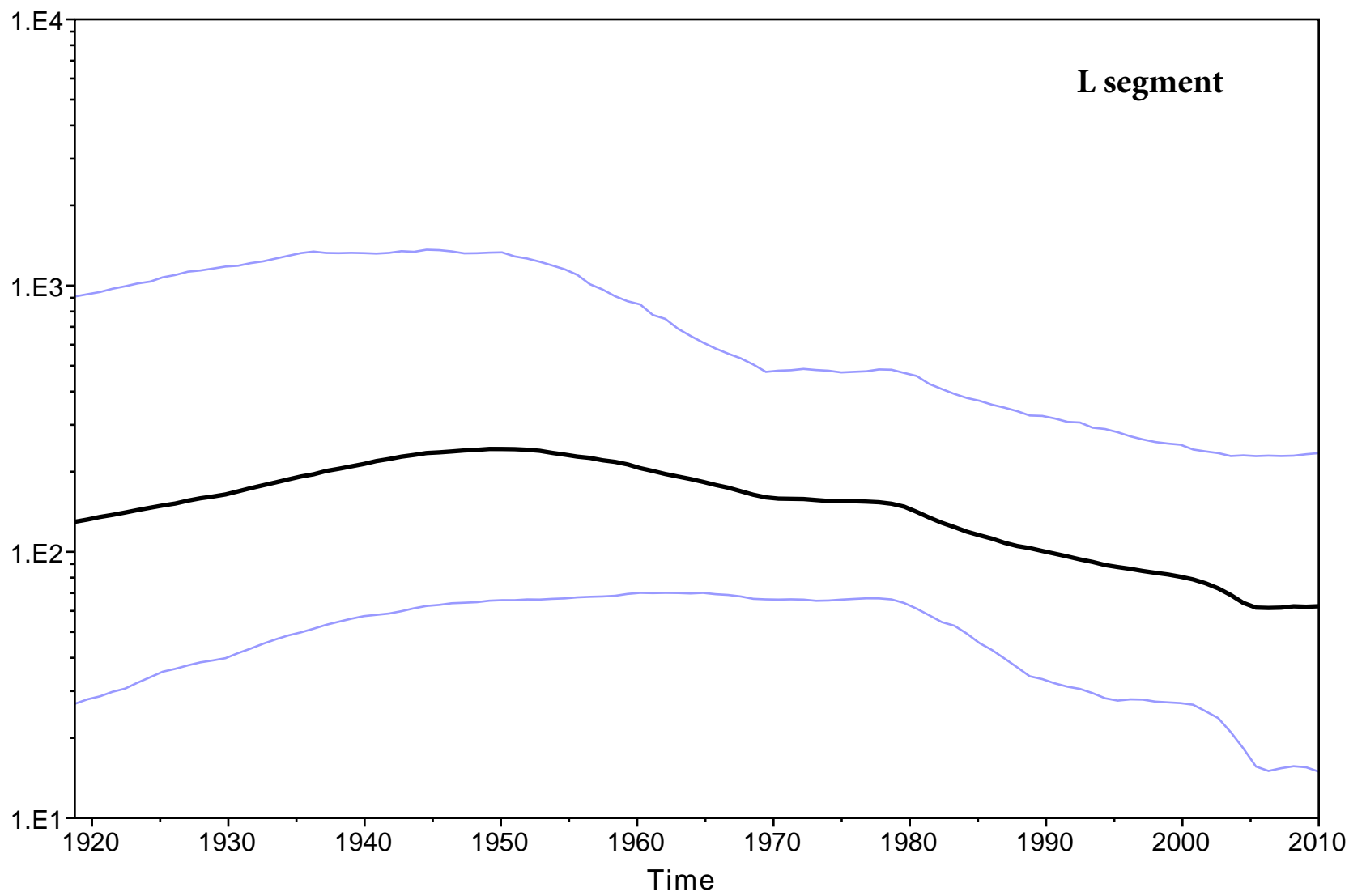

Supplement: S4 File — Blue lines show the boundaries of the 95% highest posterior density interval. (PDF) [file pntd.0005226.s004.pdf]

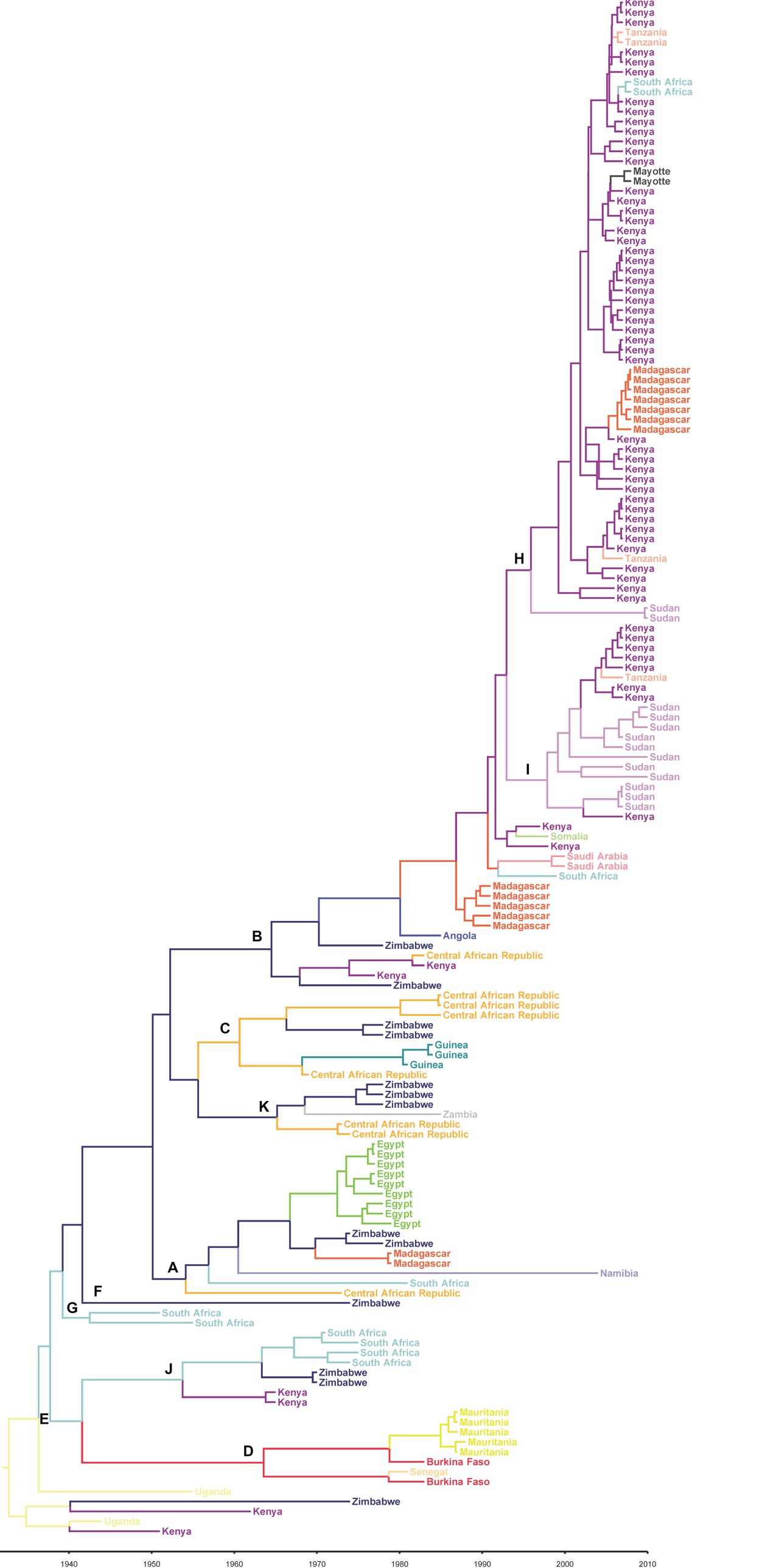

Supplement: S5 File — Country of origin is indicated by color on the tree branches and branch tips. An online version of the tree is available via https://figshare.com/s/1efd1db044bcaa9e35a9. (TIFF) [file pntd.0005226.s005.tiff]

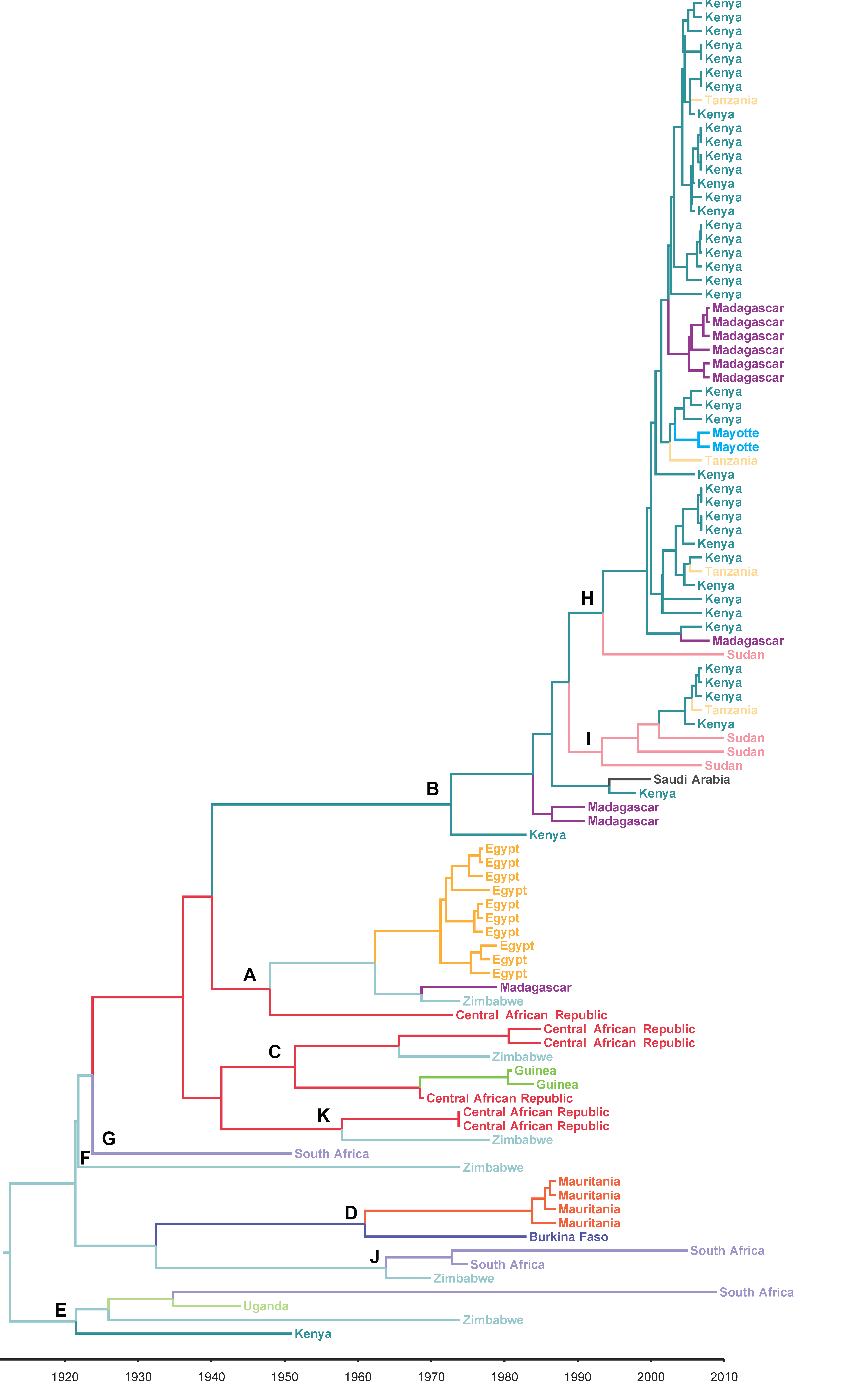

Supplement: S6 File — Country of origin is indicated by color on tree branches and branch tips. An online version of the tree is available via https://figshare.com/s/1efd1db044bcaa9e35a9. (TIFF) [file pntd.0005226.s006.tiff]

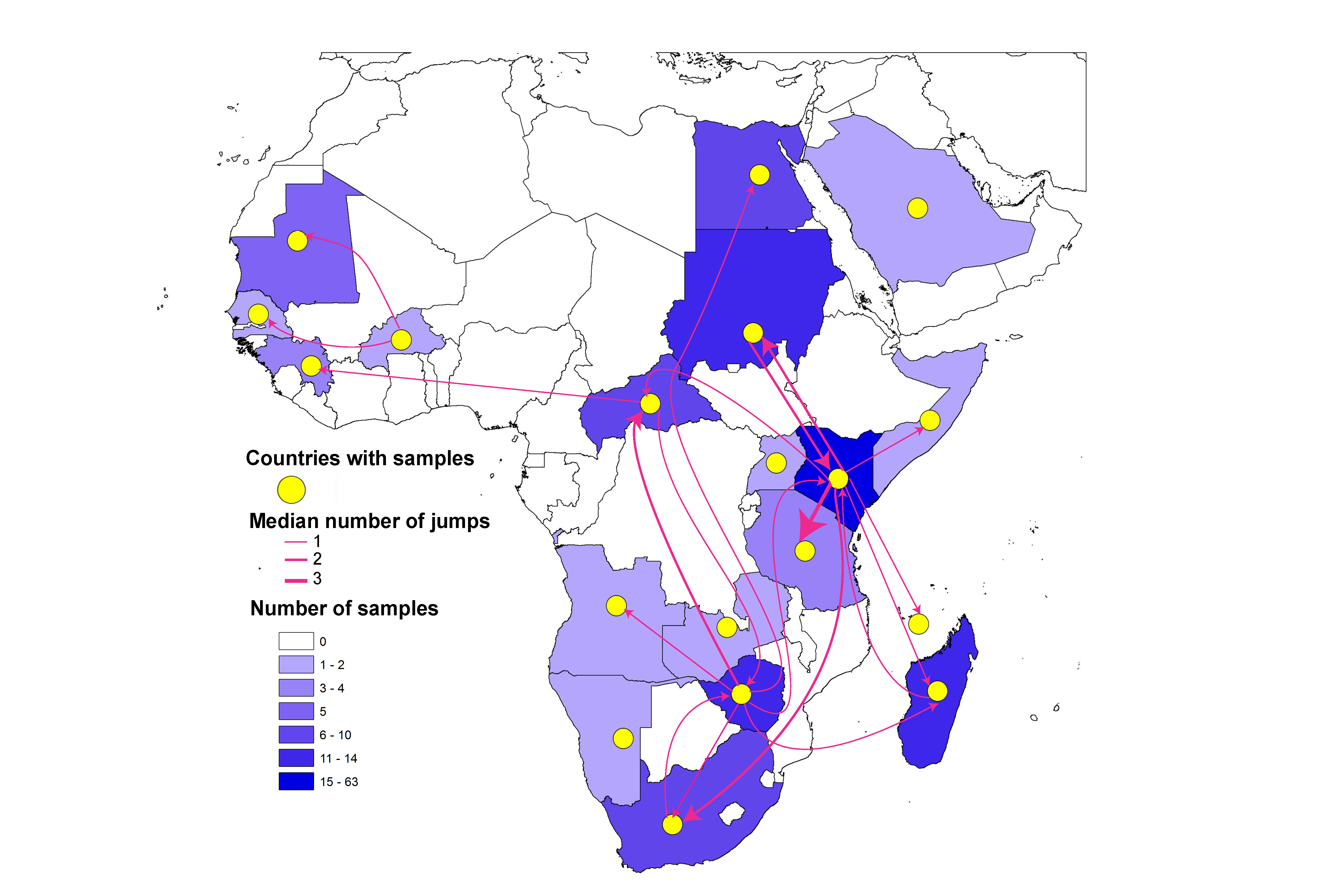

Supplement: S7 File — Connections between countries are presented as lines with arrows to refer to the direction of movement. The connected countries are countries with non-zero median transition frequencies. Line thickness identifies the median number of jumps between each country pair. (TIFF) [file pntd.0005226.s007.tiff]

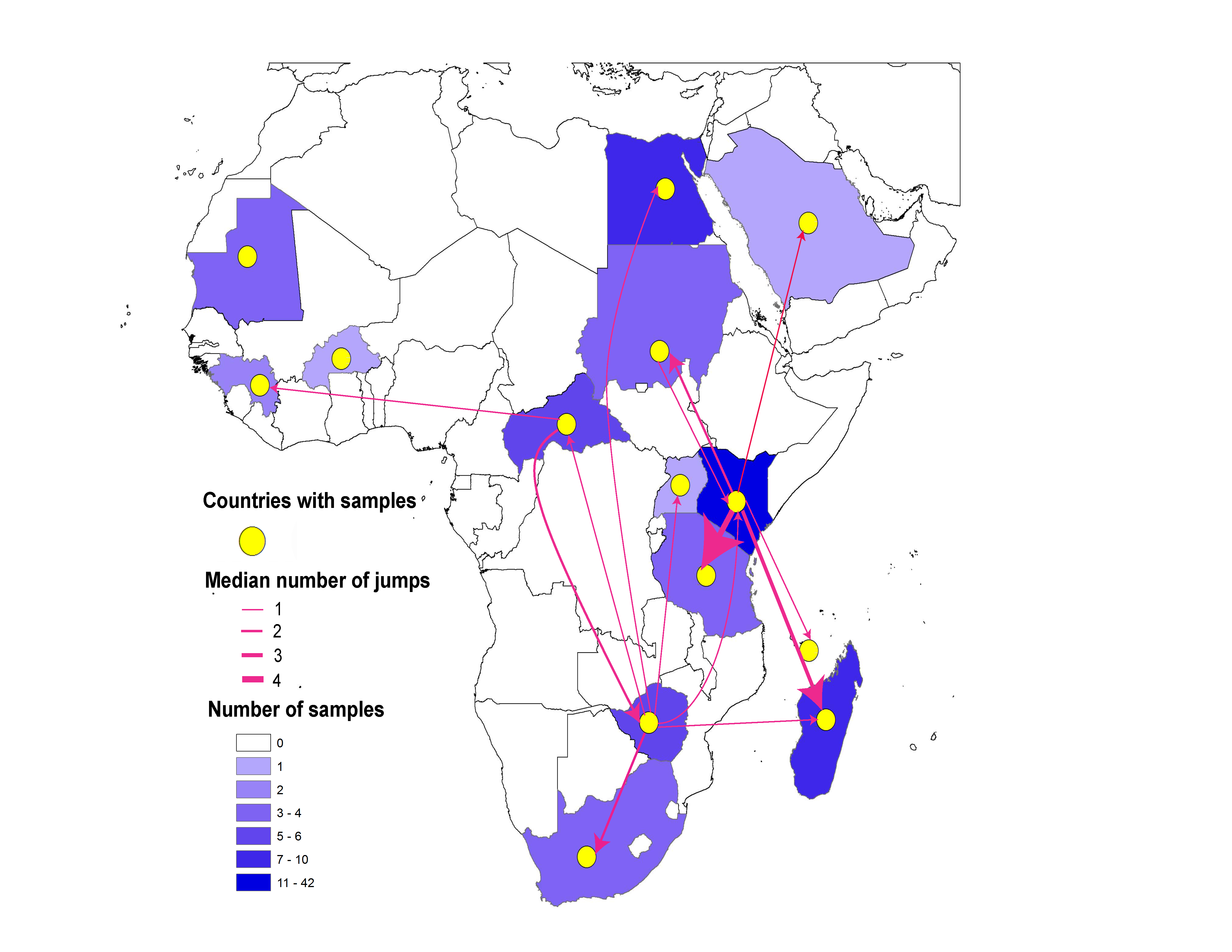

Supplement: S8 File — Connections between countries are presented as lines with arrows to refer to the direction of movement. The connected countries are countries with non-zero median transition frequencies. Line thickness identifies the median number of jumps between each country pair. (TIF) [file pntd.0005226.s008.tif]
