# Supplementary material for: Phylogeography of Rift Valley Fever Virus in Africa and the Arabian Peninsula
Source: PLoS Negl Trop Dis. 2017 Jan 9;11(1):e0005226. doi: 10.1371/journal.pntd.0005226 (PMC5221768; doi:10.1371/journal.pntd.0005226)
Supplement: S3 File — (PDF) [file pntd.0005226.s003.pdf]

| Segment | Dataset | | | |
|---|---|---|---|---|
| | Full | | * Subset | |
| | Substitution rate | TMRCA | Substitution rate | TMRCA |
| S | $3.6392 \times 10^{-4}$ | 1929 | $3.636 \times 10^{-4}$ | 1914 |
| | ($2.8114 \times 10^{-4}$ to $4.5813 \times 10^{-4}$) | (1920-1937) | ($2.058 \times 10^{-4}$ to $5.248 \times 10^{-4}$) | (1893-1935) |
| M | $3.7774 \times 10^{-4}$ | 1914 | $2.74 \times 10^{-4}$ | 1881 |
| | ($2.7391 \times 10^{-4}$ to $4.8902 \times 10^{-4}$) | (1897-1928) | ($1.965 \times 10^{-4}$ to $3.573 \times 10^{-4}$) | (1844-1907) |
| L | $2.7310 \times 10^{-4}$ | 1909 | $2.406 \times 10^{-4}$ | 1870 |
| | ($1.9289 \times 10^{-4}$ to $3.6677 \times 10^{-4}$) | (1888 -1927) | ($1.106 \times 10^{-4}$ to $4.4065 \times 10^{-4}$) | (1810-1919) |

***** Subset datasets refer to the random subsets of the original full sequences. The subset is produced by randomly removing sequences from each alignment until they contained no more than one sequence from isolates obtained in a single country during a single year.

S3 File: Substitution rate and TMRCA estimates of RVFV based on two datasets of sequences; one included all sequences in GenBank, and another one which is a random subset from the full sequences represented the downsampled dataset.
